# A composite measurement concept for monitoring cardiac function in Fabry disease

**DOI:** 10.1186/s13023-025-03895-x

**Published:** 2025-07-28

**Authors:** Pooja Nandi, Robert Ellis, Jennifer Hiros, Paul Howard, Biliana O. Veleva-Rotse

**Affiliations:** 1 Koneksa Health, New York, NY USA; 2 Koneksa Health, One World Trade Center, 285 Fulton St. 77th Floor, New York, NY 10007 USA; 3 Amicus Therapeutics, Inc., Princeton, NJ USA

**Keywords:** Fabry disease, Cardiac biomarkers, Cardiac function, Monitoring, Digital, Patient-reported outcomes, Wearable devices, Digital health technology, DHT

## Abstract

**Background:**

Fabry disease (FD) is an X-linked, multisystemic, progressive lysosomal disorder caused by *GLA* variants resulting in alpha-galactosidase A deficiency. Although cardiovascular disease is the leading cause of death in people with FD, the progression of cardiac dysfunction remains poorly understood, mainly due to a lack of clinical measurement tools for predicting cardiac progression risk over relevant timescales. New, accessible tools are needed to measure cardiac functional change and predict event risk over shorter timescales. Digital tools allow at-home, frequent data collection that could help detect elevated cardiac event risk, inform treatment and management, and support novel therapy development. Digital measures are designed, developed, and validated using recognized frameworks. We present a novel composite measurement concept aligned to established guidance that utilizes digital tools to improve the monitoring of cardiac function in FD.

**Methods:**

A targeted literature search, patient advisory board, and clinician advisory board were conducted to identify important FD signs and symptoms and the most suitable cardiac patient-reported outcomes and digital tools for concurrent remote collection of subjective and objective data.

**Results:**

The literature search highlighted a lack of FD-specific cardiac digital monitoring tools. Patient advisory board discussions and survey responses highlighted pain, gastrointestinal issues, and fatigue as important FD symptoms, and participants expressed a desire to understand how cardiac manifestations impacted these symptoms. The clinician advisory board noted a lack of specific diagnostic, monitoring, and prognosis (especially cardiac) tools in FD. The composite measurement concept was developed to capture the signs and symptoms most important to people living with FD, alongside heart-rate variability, electrocardiograms, blood pressure, and quality of life as relevant measures within the cardiac domain that can be staged in a progression model with clear group boundaries.

**Conclusions:**

Based on work completed to date, developing a composite measurement concept that utilizes digital tools to improve the measurement of cardiac function in FD is conceptually possible and aligns with the evidentiary framework for designing and building a monitoring biomarker. This composite measurement concept could be used for future analytical validation, usability, and clinical validation, seeking to capture progressing cardiac dysfunction in people living with FD.

## Background

Fabry disease (FD) is an X-linked, multisystemic, progressive lysosomal disorder caused by *GLA* variants resulting in alpha-galactosidase A deficiency, and subsequent lysosomal accumulations of glycosphingolipids and cellular dysfunction in many organs [1]. FD has a complex presentation with multiple signs, symptoms, and impacts, with the disease process beginning in infancy or even *in utero* and progressing until death [1, 2]. Cardiac involvement of FD often progresses to significant cardiac morbidity, heart failure, and sometimes death [3]. Cardiac manifestations in FD are common and can be non-specific in presentation, including but not limited to arrhythmias, left ventricular hypertrophy (LVH), myocardial fibrosis, and strokes or transient ischemic attacks [3]. Other signs and symptoms include kidney disease, gastrointestinal issues, neuropathy, and fatigue [1–3]. Symptoms – but not specifically cardiac symptoms – are covered by two recently developed FD-specific patient-reported outcome (PRO) measures, the Fabry Disease Patient-Reported Outcome (FD-PRO) [4, 5] and FABry Disease Patient-Reported Outcome-GastroIntestinal (FABPRO-GI) [6]. Disease progression is heterogeneous [7, 8] and people living with FD experience reduced functional capacity and performance [9]. According to the Fabry Registry (NCT00196742), the life expectancy of males and females with FD (58.2 and 75.4 years, respectively) markedly contrasts with that of the general population for males and females (74.7 and 80.0 years, respectively) [10].

### Leading causes of death in FD

Cardiovascular disease is the most common cause of death in people with FD; based on a 2016 systematic review of ventricular arrhythmia and sudden cardiac death in FD, cardiovascular causes accounted for 75% of all deaths [11]. In addition, an earlier article from the Fabry Registry study reported that cardiovascular disease (CVD) is the most common cause of death for both males and females living with FD (40.0% and 41.7% of all deaths, respectively) [10]. By comparison, in the US general population, diseases of the heart (per Centers for Disease Control and Prevention classification) were the most common causes of death in 2019, representing 23.1% of all deaths [12]. LVH and cardiac arrhythmia are present in 61% and 24% of patients with FD, respectively [13]. Data from other studies suggest that arrhythmias are present in 26–42% of males and 27% of females living with FD [14, 15]. Research shows that people with FD are more likely to experience certain cardiac events (myocardial infarction with non-obstructive coronary arteries) than those with hypertrophic cardiomyopathy from other causes [16].

End-stage renal disease has been shown to be a major cause of morbidity and mortality in FD [2]. Renal degeneration is well documented and can be readily measured and monitored via estimated or measured glomerular filtration rate, urinary protein:creatinine ratio, urinary albumin:creatinine ratio, and other measures [17, 18]. However, the progression of cardiac dysfunction is more heterogeneous and less well understood [19, 20]. As mentioned earlier, cardiac dysfunction may become apparent via the onset of arrhythmias or as cardiac-related clinical events [3]. Though electrocardiogram (ECG) abnormalities present early in life and progress over time, their prognostic ability for future clinical events remains unknown in FD [19]. While measures of renal decline are singular, well documented, and readily obtained [21], multiple measures of cardiac function have been observed to decline after diagnosis [3], but are usually captured during scheduled clinic visits that are typically 2–5 years apart [22].

In 2015, 41.5% (102.7 million people) of the total US population had at least one CVD, rising to a projected 45% (131.2 million people) by 2035 [23]. Based on data from 2019 to 2020, the annual direct cost of CVD in the USA is estimated as $254.3 billion [24]. People with FD comprise a portion of the total US population with CVD and are faced with a significant cost-of-care burden. Data from Europe suggest that people living with FD comprise 1% of people with hypertrophic cardiomyopathy and 0.9–1.1% of people presenting with LVH [25–27]. More accessible and affordable methods are required that enable timely intervention and reduce the number of required clinic visits for people living with FD.

### The need for a monitoring biomarker to measure cardiac functional decline in FD

**Fig. 1:**
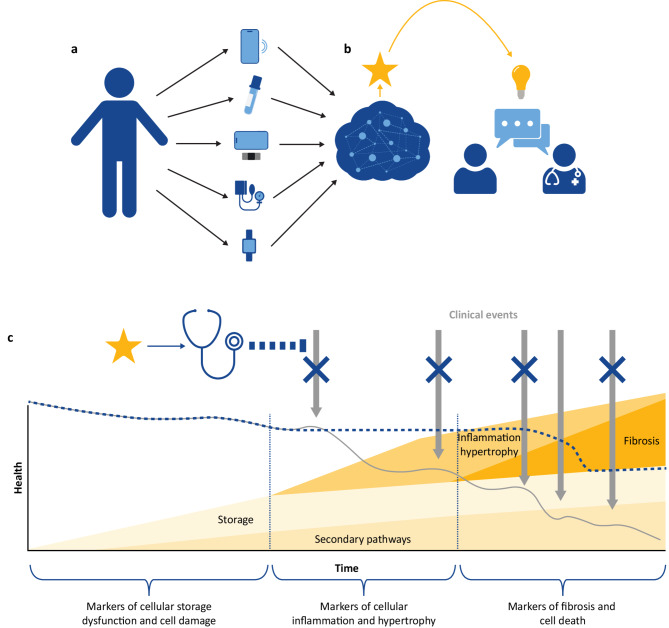
Future-state vision: patients’ signs and symptoms will be clearly understood by patients, payors, and HCPs. HCPs will have the data to link signs and symptoms to disease burden, spurring prompt management and treatment. (**a**) The individual living with Fabry disease undergoes regular multimodal assessments, including wearable data, laboratory values, vital signs, PROs, and a daily diary. (**b**) Data are transmitted regularly to a cloud-based algorithm, which integrates the information to provide an accurate snapshot of the individual’s health at that moment. The output serves as an integrated signal of both subjective experience and objective signs that the individual can discuss with the HCP. (**c**) Based on the signal and this shared decision-making approach, informed management and treatment decisions can be made, allowing for intervention prior to irreversible damage and ultimately preventing serious clinical events (arrows) and preserving health status (dashed blue line vs. solid gray line). HCP, healthcare professional; PRO, patient-reported outcome. Adapted with permission of Elsevier Science & Technology Journals, from Pieroni M et al. Cardiac involvement in Fabry disease: JACC Review Topic of the Week. *J Am Coll Cardiol*. 2021;77(7):922–36 Copyright © 2021; permission conveyed through Copyright Clearance Center, Inc.

The evidence discussed herein points to the need for new measures of cardiac functional decline in FD capable of measuring cardiac functional change and event risk in shorter timescales than current care protocols. Such measures need to be both proximal measures of cardiac function and also show convergent validity with established cardiac outcome measures. One solution is the provision of more frequent and accessible measures using digital tools that enable patient measurement at home. This approach is a step toward the ultimate goal of objectively measuring subjective impacts of disease so that healthcare providers and people with FD can engage in informed, shared decision-making around disease management and treatment (Fig. 1).

FD has heterogeneous and highly individual progression patterns and currently does not have a defined generalized, stepwise progression stage model [7, 28], unlike other diseases such as Parkinson’s disease, multiple sclerosis, or congestive heart failure. Progression models define known disease stage groups and support the demonstration of construct validity and response analysis, which are typical aspects of clinical validation. Cardiac FD progression may be more challenging to fit into linear models given the multiple tissue types involved, including nervous, muscular, and vascular tissues [3]. The X-linked nature of FD poses additional challenges to the development of a one-size-fits-all approach.

While the cardiac “red flags” by decade of life in FD have been defined [3, 29], the presence of these flags does not support timely prognostic intervention but rather an “after the fact” treatment approach, at which point damage is irreversible. More frequent disease measurement is needed to support efficient novel therapy development, detect elevated cardiac event risk, and ensure appropriate management, monitoring, and treatment decisions, with the ultimate goal of preventing clinical events (Fig. 1).

While the pre- and post-LVH diagnostic boundary is only one of several cardiac red flags in FD, the evidence of statistical differences in multiple cardiac measures at this boundary lends itself to use as a known group definition for novel measurement development. Demonstrating clear differences between cardiac measures collected before and after LVH in people living with FD versus the general population will further inform the current understanding of FD progression.

Progression models must be powered appropriately for both males and females with FD to account for known sex-based differences in cardiac disease progression [19, 20]. Recent research suggests that female cardiac disease progression may occur even faster as FD progresses, even though ECG measures in the early stages of their disease may appear “super normal” [19]. This lends credence to the theory that FD manifests itself differently, not just at different rates, in males and females.

### Evidentiary framework, V3, and digital health technology (DHT) guidance for designing and building a monitoring biomarker

**Fig. 2:**
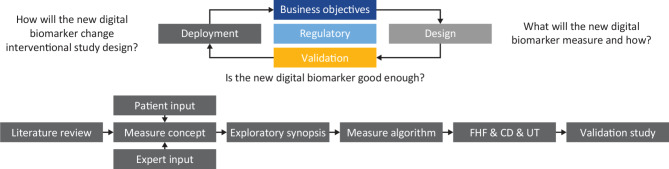
Developing a novel digital biomarker. CD, cognitive debriefing; FHF, formative human factors study; UT, usability testing

To effectively design, build, and validate a novel digital biomarker to meet regulatory standards, clinical association and concept development work must be completed prior to analytical validation, usability testing, and clinical validation for application in clinical practice (Fig. 2). Novel digital measures are developed using recognized frameworks such as the evidentiary framework [30], verification, analytical validation, and clinical validation (V3) framework [31], software as a medical device [32], or the DHT guidance [33]. The frameworks outline consistent terminology and best practices for validation of digital measurement tools across different settings and intended uses. Once the need for, or clinical relevance of, the new digital tool has been established, a framework helps to define what the new tool should measure and how. Through analytical validation, it must be shown that the tool replaces existing industry gold standards. At this point, usability and clinical validation of the new tool must be established within the intended population [31–33]. This could include a formative human factors study, conducted to formally ascertain the safety and usability of a digital toolkit in the intended population and context of use, and cognitive debriefing and usability testing to evaluate whether patients can complete digital tools (e.g. electronic patient-reported outcomes [ePROs]) without additional burden or reduction in comprehension compared with paper source versions [34].

### Applying the framework for designing and building a monitoring biomarker in FD

Our goal is to determine if it is possible to define a composite measurement concept that utilizes new and established digital measures to improve the measurement of cardiac function in FD. This paper outlines the clinical association and concept development work that has been done to date with US-based, English-speaking patients and globally based clinicians to design a novel biomarker to meet industry standards. The work outlined can be used as a basis for future analytical validation and subsequent usability, iteration, and clinical validation of the composite cardiac monitoring biomarker in people with FD who live in other countries and speak other languages.

## Methods

### Targeted literature search

A targeted literature search was conducted to identify cardiac and non-cardiac signs and symptoms related to FD and cardiac PROs, and other related outcome measures. The targeted literature search was intended to inform the use of patient diaries in FD, the current understanding of the FD progression model, and measures of interest. Table 1 shows sample searches conducted using PubMed as part of the targeted literature search.

**Table 1:** Sample search terms used across pubmed with counts and a high-level summary of results

Sample search term	No. of results	Initial notes
((Fabry) AND (cardiac))	1,657	Cardiac aspects of Fabry disease
((Fabry) AND (cardiac OR heart))	1,657	Heart as a synonym for cardiac
((Fabry) AND (cardiac OR heart) AND Biomarker)	136	Biomarkers in Fabry disease where cardiac is also mentioned
((Fabry) AND (cardiac OR heart) AND biomarker AND study)	80	Studies referencing biomarkers in Fabry disease where cardiac is also mentioned

### Patient advisory board

Two remote patient advisory board sessions were conducted via Zoom^®^ with nine US-based, English-speaking patients diagnosed with FD and of mixed sex and age (four males, five females; 18 years and older; approximate age range, 30–70 years). These sessions were intended to understand patients’ perspectives, qualitatively assess signs and symptoms of importance, and determine patients’ willingness to use and tolerate proposed sensors/wearable devices, to complete an at-home patient diary, and to complete ePROs. A post-session follow-up survey was completed by seven of the attendees.

### Clinician advisory board

Two clinician advisory board sessions were conducted via Zoom^®^ with eight clinical experts in FD practicing in Asia (Korea), North America (US), and Western Europe (countries included Finland, Italy, Switzerland and the UK). Clinician advisory board members were selected based on clinical expertise in cardiac aspects of Fabry disease, involvement in peer-reviewed publications on the topic, and on being representative of a broad range of countries and clinical settings. The number of participants was chosen to ensure a broad range of experiences while allowing for interactivity and meaningful contributions from each in the time allotted for the discussions. The clinician advisory board sessions were intended to obtain expert feedback on the case for a cardiac monitoring biomarker and the proposed composite measurement concept, and to share feedback collected to date from people living with FD. Topics were raised by the session moderator, with all clinicians given the opportunity to provide their insight. All feedback shared by each clinician was reiterated live by the moderator to allow other clinicians to raise concerns or provide additional suggestions. Any disagreements were discussed live between clinicians in-session until all attendees agreed on the summarized final output of key recommendations.

### Measurement concept development

A measurement concept was developed based on the targeted literature review, as well as patient advisory board and clinician advisory board feedback.

## Results

### Targeted literature search

Review of studies on PubMed identified more than 70 papers published between 2000 and 2022 for analysis. While there were many papers on FD in general, analysis of the literature search findings across a range of categories (general signs and symptoms, cardiac signs and symptoms, cardiac PROs, other outcome measures, patient diaries, FD progression models, and digital monitoring tools) found none relating to digital cardiac monitoring biomarkers in FD.

#### Signs and symptoms

There are many symptoms of interest in FD. Three main measures, the FD-PRO [4, 5], Mainz Severity Score Index (MSSI) [35, 36] and the Fabry Stabilization Index (FASTEX) [37, 38], have been developed for, and are often used in, adults living with FD. However, these tools do not specifically focus on cardiac complications.

The FD-PRO, published in 2021, is a frequently used instrument to assess patient experience in FD [4]. Development of the FD-PRO included a survey of signs and symptoms in people living with FD, the results of which showed that neuropathic pain, temperature intolerance, energy difficulties, hearing/vision impairment, and gastrointestinal symptoms were most frequently mentioned, with neuropathic pain, stomach pain, burning pain, and fatigue as the most severe symptoms of FD overall [4]. Cardiac and blood pressure (BP) symptoms were less frequently reported but ranked reasonably high in terms of severity and in how bothersome patients considered them to be [4]. However, clinicians who completed the survey flagged cardiac and renal disease as focus areas since they collectively account for 75% of morbidity or premature deaths [39, 40]. Limitations of the FD-PRO include the inability to attribute changes in signs and symptoms to underlying disease or treatment side effects, an inability to differentiate symptom patterns between males and females, and the significant number of items presenting a notable participant burden [4].

The MSSI, published in 2004, is also used to assess the progressive nature of FD across four domains: general, neurological, cardiovascular, and renal [35, 36]. The MSSI ranks cardiac and renal signs and symptoms as the highest causes of FD morbidity [35, 36]. Though the MSSI has been validated previously in FD as a disease severity scoring system, there are limitations to its repeated use. One of these limitations is the reliance on laboratory and clinical measures that require in-person hospital visits [35]. While it does include some subjective symptoms, these are few and scored for presence/absence, not for their degree of impact on the individual.

FASTEX, developed in 2016, is another instrument frequently used to monitor disease progression in FD across three domains: nervous, renal, and cardiac [38]. However, FASTEX has limitations, including its subjective and retrospective nature, operational challenges with online completion, and lack of automatic calculations of weighted scores [37].

#### Cardiac signs and symptoms

Cardiac measures of interest in FD change with disease progression. In the early years of disease progression, the PQRST waveform contains features indicative of FD [41]. Research suggests that the T/R amplitude ratio, (T_onset_–T_peak_) / (T_peak_–T_end_) [in ms], and T-wave amplitude differentiate patients with prodromal LVH from healthy individuals, and also that numerous ECG intervals and amplitudes differentiate prodromal LVH from diagnosed LVH [42]. However, it is not known how these ECG measures change over time and what their prognostic significance may be.

Single-lead clinical-grade ECG devices are available for home use (e.g. those developed by Boston Scientific Cardiac Diagnostics, Inc. [43] or Vivalink [44]) and may therefore meet the requirement for more frequent measurement of cardiac function. One case study reports an asymptomatic female who presented with abnormal cardiac imaging and no other clinical manifestations of FD besides abnormal ECG, which revealed short PR intervals with increased QRS voltages and ST segment/T-wave alterations; upon further investigation, she was found to have substrate deposition in cardiomyocytes, an early pathological sign of FD [45]. Research suggests that monitoring the rate of change in ECG parameters can be utilized to measure disease progression, inform treatment, and evaluate its effectiveness [19]. When monitoring changes in ECG during adult life, however, it is critical to consider the apparent age and sex dependence of ECG deviations in FD [19].

Heart-rate variability (HRV) is an overall indicator of heart health and a predictor of heart-related mortality after a myocardial infarction or in people with chronic heart failure compared with the general population [46]. Research suggests that HRV may be a useful biomarker for FD from an early age, though differences are observed between males and females [47]. HRV is also associated with the progression of kidney disease [48] and atrial fibrillation [49], which is a type of arrhythmia (a symptom of FD) and is an indicator of stroke risk [41, 50].

Hypertension is observed in people living with FD [51]. However, hypertension is also common in the general population and may be non-specific with respect to FD-related cardiac disease.

#### Cardiac PROs

In addition to monitoring objective measures, outcome measures can capture additional context about an individual’s cardiac health status at various time points. The Minnesota Living with Heart Failure Questionnaire (MLHFQ) and the Kansas City Cardiomyopathy Questionnaire (KCCQ) are heart disease outcome measures. Both questionnaires are validated [52, 53], qualified for use as a medical device development tool [54, 55], and commonly used in interventional and care management trials, including in FD [56–58]. Both measures demonstrate the ability to assess fine-grained changes in quality of life in different heart diseases [59]. The main difference between the two outcome measures is the number of items (MLHFQ: 21; KCCQ: 23) and the length of the recall period (MLHFQ: 4 weeks; KCCQ: 2 weeks) [54, 55, 59].

#### Other outcome measures

Fatigue is a commonly reported symptom of FD [2]. Fatigue is a multifaceted concept with physical, cognitive, and social impacts, and is often associated with chronic illness [60]. Fatigue could be related to many frequently reported FD symptoms, including cardiac disease, renal disease, pain, and gastrointestinal problems. The many compounding variables contributing to fatigue make it difficult to measure precisely. The Functional Assessment of Chronic Illness Therapy – Fatigue (FACIT-F) is a commonly used questionnaire that comprises 40 questions across five domains with a 7-day recall period [61]. While FACIT-F was developed for use in oncology [61], it has been frequently used across many diseases. The Patient-Reported Outcome Measurement Information System Short-Form 13 (PROMIS SF-13a) is an abridged version of the FACIT-F adopted by the PROMIS Health Organization, comprising 13 items with a 7-day recall period [62].

People living with FD have a lower quality of life and reduced functional status compared with the general population, and this begins in childhood [63–65]. Functional status is an individual’s ability to perform normal daily activities to meet basic needs, fulfill usual roles, and maintain health and well-being. Concept models have been developed to describe the various contributions to functional status and their impact on quality of life [66, 67]. The EuroQoL 5 dimensions, 3 levels (EQ-5D-3L) [63] and EuroQoL 5 dimensions, 5 levels (EQ-5D-5L) [68] have been used to assess quality of life in FD. Both EQ-5D questionnaires assess health in five dimensions (mobility, self-care, anxiety/depression, usual activities, and pain/discomfort), with 3 L/5L referring to the number of response levels, and involve same-day recall [69, 70]. The latter aspect is advantageous for comparison with objective measures of cardiac function that may be deployed at flexible and frequent timescales.

#### Patient diaries

Patient diaries are routinely used in care management situations to track an individual’s compliance with medication and monitor recovery, such as tracking daily or less-frequent changes in symptoms, and to support care conversations with physicians. Diary data support analysis of objective measures captured at a higher frequency than PROs and segmentation of short-timescale changes resulting from background disease progression [71].

#### FD progression models

Previous non-FD studies have shown that the pre- and post-LVH diagnostic boundary supports the identification of statistically significant differences in HRV [72] and ECG waveform properties [73]. There is a significant reduction in HRV (measured by the standard deviation of the normal-to-normal interbeat interval) in people with LVH secondary to hypertension or aortic valve disease compared with asymptomatic controls; combining data from both groups demonstrated an inverse relationship between HRV and left ventricular mass (LVM) index [72].

In people living with FD, a strong association between LVM progression and the risk of clinical events has recently been established [74]. Indeed, progression in LVM index may already occur in childhood/adolescence [47]. In addition, people living with FD may experience increases in BP (for example from hypotension to hypertension) that accompany FD progression and may contribute to major organ damage [75]. While the pre- and post-LVH diagnostic boundary is only one of several cardiac red flags in FD, the evidence of statistical differences in multiple cardiac measures at this boundary lends itself to use as a known group definition for novel measurement development.

Furthermore, when analyzing FD progression, the literature emphasizes the need to consider sex-based differences [76]. A study conducted by Niemann et al. demonstrated that, in contrast to male patients, development of fibrosis in females does not necessarily require the occurrence of LVH [76]. Additionally, since the literature search was conducted, analysis of HomeCageScan data from male and female rats with FD (Rat Genome Database symbol: *Gla*^em2Mcwi^) has found differences in their behavior relative to wild-type rats of the corresponding sex [77]. Overall, female FD rats in particular spent less time caring for themselves, moving around, and exploring than wild-type rats, consistent with reductions in movement and daily activity observed in people living with FD. Additionally, wild-type female rats were observed to be more active than wild-type male rats [77], pointing to the need for sex-based comparisons in people living with FD to account for noticeable sex-based differences.

The literature search revealed a study conducted in 2022 by Orsborne et al. that developed and internally validated a risk prediction model capable of predicting a 5-year risk of adverse cardiac outcomes for people living with FD utilizing age, LVM index and dispersion of myocardial relaxation time (T_1_) [78]. Despite the model’s potential for predicting adverse cardiac outcomes, its follow-up timescale of ~ 4.5 years is significantly longer than timescales typical of cost-effective interventional trials.

#### Digital monitoring tools

**Fig. 3:**
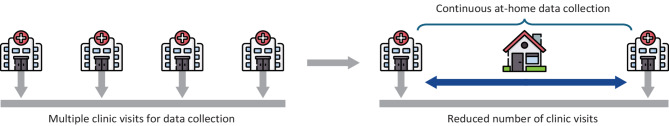
Potential benefits of digital monitoring tools Digital monitoring tools enable collection of high-frequency health data and reduce the need for multiple clinic visits

The use of wearable devices, including sensors along with software applications on smartphones or tablets, in clinical trials has drastically escalated in recent years. Wearable devices enable the collection of high-frequency health data as people go about their daily lives and reduce the need for multiple in-clinic visits [79]. In addition to reducing patient burden, vital sign data captured in the clinic as per standard clinical trial procedures only capture a snapshot of a patient’s overall health at a single point in time (Fig. 3). This presents a challenge, specifically in FD, as it is unclear how and over what timescale ECG perturbations correlate with clinically apparent cardiac manifestations.

Wrist-worn wearables, such as the Empatica EmbracePlus (Empatica, Cambridge, MA, USA) [80], can passively capture activity, walking, and sleep measures at multiple time points. The Empatica EmbracePlus device can also capture cardiac measures as measured by its photoplethysmography sensor including normal-to-normal interbeat interval, heart rate per 10-second epoch, and HRV time and frequency domain measures. The Empatica EmbracePlus is a US Food and Drug Administration (FDA)-cleared, EU Conformité Européene (CE)-marked sensor that transmits data via Bluetooth or USB through a smartphone-installed application.

Single-lead, clinical-grade ECG devices, such as the Vivalink VV330 (Vivalink, Campbell, CA, USA) [44], are also available for home use to capture frequent measurements of cardiac function. The Vivalink VV330 is a chest-worn patch that captures a raw 5-minute ECG trace, interbeat (RR) interval, PQRST waveform measures, and HRV time and frequency domain measures. The chest-worn patch is FDA-cleared, has an EU CE mark, and transmits data via Bluetooth.

The Withings BPM Connect (Withings, Paris, France) is a clinically validated, FDA-cleared, and EU CE-marked BP monitor that captures both systolic and diastolic BP [81]. The performance of the device was validated in the general adult population in a clinical study conducted following a protocol developed by the European Society of Hypertension, the Association for Advancement of Medical Instrumentation in the United States, and the International Organization for Standardization [82]. The device uploads data via Wi-Fi and Bluetooth through a smartphone-installed application.

A smartphone can be used along with wearable devices to enable remote data upload while capturing additional measures, such as activity and mobility, through its built-in sensors. Patient diaries and PROs can be built into smartphone applications to capture patients’ perceived health status at more frequent timescales than traditional in-clinic visits, and provide context to device-collected measures [79].

### Patient advisory board

#### Remote portion

There was unanimous agreement among the patient advisory board members that, given the multiple signs and symptoms of FD, it is difficult to pinpoint the main symptom that negatively impacts daily life. Based on in-session discussions and responses to the follow-up survey, pain, gastrointestinal issues and fatigue were highlighted as symptoms of importance. Participants also expressed a strong desire to know if cardiac events are occurring, so that they know they are not in a “danger zone” even if they feel unwell, and fear cardiac-related sudden death. Patients also expressed concern about not knowing the extent to which cardiac signs and symptoms impact their daily life. Additional dialogue revealed sex differences in the desire to collect and document measures. Firstly, women, in particular, frequently reported feeling dismissed or struggling to be taken seriously by clinicians and their families when discussing their FD pain and fatigue symptoms, and this even had the effect of making them doubt their own experiences. There was an overall strong consensus among the women regarding the desire to record measures that would help justify earlier intervention and support conversations with clinicians. Although men did not report similar experiences, they expressed a strong desire to track objective measures of their disease progression over time to inform them and help them to actively participate in care decisions. Secondly, women reported lifestyle changes, reporting longer periods of time than men when they were unable to go on walks or leave the house.

#### Follow-up survey results

**Fig. 4:**
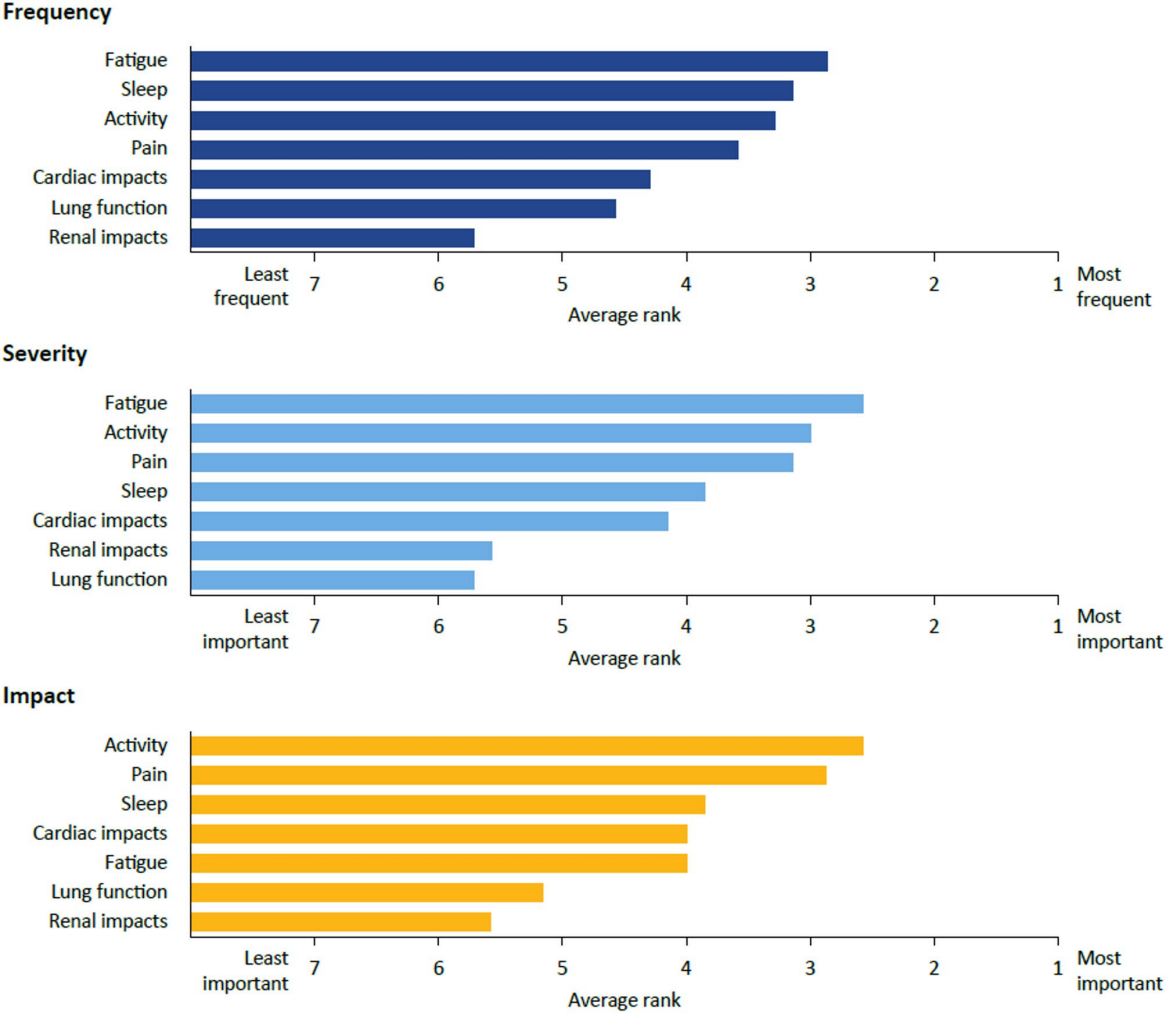
Frequency, severity and impact of Fabry disease signs and symptoms as ranked by survey participants Seven of the participants in the patient advisory board completed a follow-up survey in which three separate questions prompted them to “rank [their] Fabry signs and symptoms from most important (1) to least important (7)” in terms of daily frequency, severity, or impact

Figure 4 shows aggregated responses on the ranking of signs and symptoms in the follow-up survey. On average, fatigue and impacts on sleep were the most frequent, fatigue and impacts on activity were the most severe, and impacts on activity and pain were the most impactful. In response to a separate question, almost half of the participants (43%) reported that FD prevented them from doing normal activities on more than half of the days in the previous month. One participant rephrased the impact to state, “Fabry does not affect how I normally do things, it affects the things I want to do [consider doing].”

**Fig. 5:**
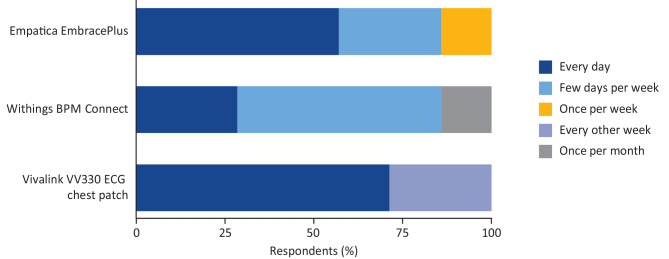
Survey participants’ preferred wear burden for monitoring devices Seven of the participants in the patient advisory board completed a follow-up survey in which they were asked about how often they would be willing to wear particular devices. ECG, electrocardiogram

Participants were also presented with an overview of the use of digital measures in clinical trials, a proposed study design, candidate measures, devices, and a proposed measurement schedule. Figure 5 summarizes patient wear tolerance of presented devices in consideration for measurement of cardiac and additional FD symptoms at home. All participants were willing to wear the Empatica EmbracePlus wrist device at least once a week and most were willing to use the Withings BPM Connect BP cuff at least a few days a week. While the majority of participants were willing to wear the Vivalink VV330 ECG chest patch every day, two preferred to wear it only every other week.

**Fig. 6:**
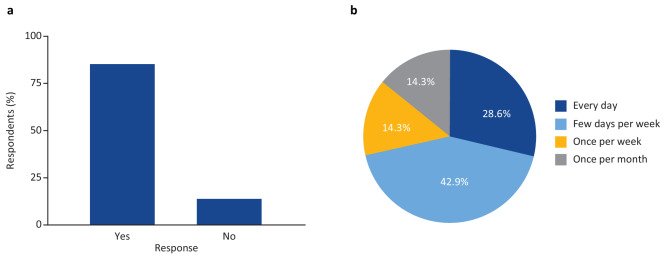
Survey participants’ attitudes to tracking Fabry disease symptoms in a diary Seven of the participants in the patient advisory board completed a follow-up survey in which they were asked (**a**) whether tracking symptoms in a patient diary would be useful to them as patients and (**b**) how often they would be willing/able to complete a patient diary of their symptoms that they may share with their clinician

Additionally, patients were asked about completing established questionnaires and the use of diaries to record FD-related symptoms at home. Overall, patients indicated a willingness to spend up to 15 min a few times a week completing questionnaires. Patients noted that in their experience, questions are sometimes repeated across different questionnaires and that this should be avoided when possible. The majority of participants reported that completing an FD symptom diary would be useful (Fig. 6A). Patients were willing to spend up to 15 min completing a patient diary, with the majority willing to do this a few or more days a week (Fig. 6B). Many participants also shared that they regularly maintain a diary to support conversations with their clinician.

### Clinician advisory board

Clinical experts emphasized the challenges surrounding frequent clinician inability to identify FD, monitoring, and prognosis of the disease. There was overall agreement on the importance and need for the development of a monitoring cardiac biomarker to support CVD therapies specific to FD, care management situations, clinical conversations with patients, and remote monitoring.

Clinical experts recommended that the measurement concept consider all indicators of LVH as known group boundaries, as well as disease onset and progression differences with age and sex. Furthermore, experts emphasized the importance of investigating cognitive (brain fog, anxiety, depression, etc.), functional, and sleep impacts of FD. There was shared support for utilizing cardiac outcome measures to assess cardiac status and provide context to objective measures, with a strong preference for inclusion of the KCCQ over the MLHFQ in the measurement concept.

### Composite measurement concept

**Fig. 7:**
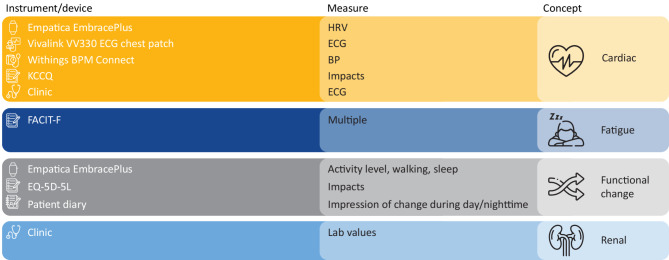
Candidate composite at-home measurement concept. BP, blood pressure; ECG, electrocardiogram; EQ-5D-5L, EuroQoL 5 dimensions, 5 levels; FACIT-F, Functional Assessment of Chronic Illness Therapy – Fatigue; HRV, heart rate variability; KCCQ, Kansas City Cardiomyopathy Questionnaire

The composite at-home measurement concept developed as a result of the targeted literature search, and patient advisory board and clinician advisory board feedback is outlined in Fig. 7. After establishing eligibility and consent for enrollment, people living with FD would additionally be assessed for caffeine, alcohol, and smoking habits and have their diagnosis of FD and whether they are pre-or post-LVH diagnosis confirmed.

## Discussion

The high unmet medical need, heterogeneity, and subsequent lack of understanding of the progression of cardiac dysfunction in FD points to the need for new digital measures capable of measuring cardiac functional change and event risk, more frequently and conveniently for people living with FD, and at shorter timescales than currently available. Such measures will enable the development of a defined global progression stage model that will allow for earlier diagnosis, continuous monitoring, and timely intervention for those living with FD.

As outlined earlier, to effectively design, build, and validate a novel digital biomarker that meets regulatory standards, clinical association and concept development work must be completed, followed by analytical validation, usability testing, and clinical validation (Fig. 2).

We present a novel composite measurement concept aligned with established guidance that utilizes digital measures to improve the measurement of cardiac function in FD. In alignment with regulatory standards, such as the FDA PRO guidance [83], the composite measurement concept should include signs and symptoms most important to patients, as well as measures of relevance that can be staged in a progression model with clear group boundaries. Additionally, the concept should include both objective and subjective methods for capturing changes in these signs, symptoms, and measures at frequent timescales while posing minimal burden to patients.

### Signs and symptoms

The targeted literature search revealed pain and fatigue as the most severe symptoms in FD, with pain, breathing difficulties, fatigue, and constipation being the most bothersome [4]. Although cardiac and BP symptoms are less frequently reported, they also rank highly in terms of severity and in how bothersome patients consider them to be. It may be relevant to note that while concept elicitation for the development of the FD-PRO included cardiac impacts [4], cardiac impacts are ranked lower than pain and activity impacts. This is despite cardiac disease being the primary cause of death for people with FD [10, 84]. The dissonance between cause of death and patient concept elicitation suggests that people living with FD may not be aware of progressive cardiac impacts on their functional status given the multisystemic nature of FD. It may also be the case that, because cardiac disease does not manifest in the same direct way as pain, fatigue, or gastrointestinal problems, patients are less aware of cardiac disease progression. Furthermore, adults with FD frequently report exceptionally elevated high-density lipoprotein cholesterol levels, or dyslipidemia [85].

Patient advisory board discussions and follow-up survey responses further support the importance of pain, gastrointestinal problems, and fatigue in FD. Participant feedback suggests that while people living with FD are aware that they have FD-related cardiac signs and symptoms, they are not aware of how these signs and symptoms impact their daily life. Participants also expressed strong willingness to better understand how their symptoms affect their daily life and how they are related to objective measures of their health to better support clinician conversations and appropriate clinical management.

The strong desire and need expressed by participants to better understand their symptoms and impacts supports the first step of designing a novel digital biomarker to meet industry standards: clinical association or establishing the need for the new digital biomarker. The signs and symptoms identified in the targeted literature search and patient advisory board were used to inform the second step in the process, concept design, which involves establishing what the new digital biomarker is looking to measure. The measurement concept (Fig. 7) was subsequently updated to include cardiac, fatigue, and functional status domains of interest based on the information gathered.

### Significance of the LVH boundary

The development of LVH is one of several phases in the cardiac decline observed in FD, is well documented in the literature, and is relatively distinct [3, 86]. Well documented and distinct boundaries are ideal for novel measurement development and subsequent validation as they provide an anchor that enables new and existing measures to be analyzed collectively. In such situations, the validity and size of change in the new measure and existing measures can be determined. Such boundaries are also present in other diseases, such as the Hoehn and Yahr progression stages in Parkinson’s disease [87], which have been used to define changes in Movement Disorder Society Unified Parkinson’s Disease Rating Scale scores [88].

As identified in the targeted literature search, the risk prediction model developed by Orsborne et al. [78] includes follow-up timescales of approximately 4.5 years, which is significantly longer than timescales typical of cost-effective clinical trials. Another risk prediction model by Meucci et al., which involves stratification based on echocardiography parameters (and was published after the targeted literature search cut-off), followed patients for a median of 5.7 years [89]. Utilizing continuous digital at-home measures in conjunction with regular clinical cardiac monitoring protocols may help address gaps in the existing predictive model by providing higher-frequency inputs. These at-home measures may have the additional benefit of lower time and cost burdens on the patient and healthcare system.

Previous non-FD studies have shown that the pre- and post-diagnostic boundary supports identification of statistically significant differences in HRV [72] and ECG waveform properties [73]. Proximal cardiac measures of interest in FD have also been found to change with disease progression [41, 42, 75, 90]. The literature and expert conversations emphasized the importance of considering all indicators of LVH as group boundaries when developing a measurement model for disease progression. Measurement of the PQRST waveform and HRV provides insight into cardiac function in FD and may show statistically significant differences around key progression events [41, 42, 47]. Both the literature and experts highlighted that HRV is an overall indicator of cardiovascular health and a predictor of mortality, for example after myocardial infarction or in people with heart failure [46]. HRV may be a useful biomarker for FD from an early age, but any further investigation must consider potential differences between males and females as seen, for example, in a small study in children and adolescents with FD [47].

The measurement concept (Fig. 7) was developed to include HRV, ECG measures, BP, and impacts on daily life as measures of interest within the cardiac domain based on the targeted literature search and conversations with experts. Inclusion of these cardiac measures meets the concept design criteria in the general framework of designing a novel digital biomarker as per industry standards, i.e. defining what the biomarker is seeking to measure (Fig. 2).

### Sex-based differences

The targeted literature review and expert sessions emphasized the need to consider differences between males and females in FD progression, specifically considering that LVH almost always precedes fibrosis in males but not necessarily in females. Analysis of the behavior of male and female FD rats compared with wild-type rats has also demonstrated both sex and genotype differences in several home cage behaviors, mirroring differences in lifestyle changes typically observed in people living with FD [77]. Female patient advisory board participants reported lifestyle changes, with longer periods of time when they were unable to go on walks or leave the house than their male counterparts. Both patient testimony and animal studies indicated sex-based differences in FD manifestation. In addition, female patients with FD reported different goals for gathering objective measures of their subjective experience compared with male patients, suggesting both biological and/or clinician discrepancies in terms of management of their disease.

The strong shared desire to monitor disease progression across both males and females further supports the clinical association step of designing a novel digital biomarker (Fig. 2). Future analytical validation of the composite measurement concept must consider sex-based differences when defining patient cohort criteria and during analysis of collected measures.

### Data collection

The concept development step of designing a digital biomarker requires establishing not only what the biomarker is intended to measure, but also how (Fig. 2).

#### At-home wearables

Single-lead, clinical-grade ECG devices are available for at-home use and may meet the requirement for more frequent measurement of cardiac function. Indeed, the Vivalink VV330 was well received by the patient advisory board (Fig. 5), supporting its use in the proposed measurement concept (Fig. 7). Similarly good support was apparent for wearing devices to collect BP (Withings BPM Connect device), HRV, activity, walking, and sleep measures (Empatica EmbracePlus) across the cardiac- and functional-status domains of the measurement concept (Fig. 5). Passively collecting measurements at home should significantly reduce the burden to patients of frequent in-clinic visits. It will also enable collection of higher-frequency measures at times of day relevant to patient experience to build a more granular model of the progression of cardiac disease and fulfill the requirement of the concept development step of building a digital biomarker as per industry standards (Fig. 2).

#### Patient diaries and ePROs

Feedback from experts showed a strong preference for inclusion of the KCCQ over the MLHFQ in the measurement concept to capture changes in cardiac impacts over time in conjunction with device-collected measures. The 14-day recall period of the KCCQ, however, is too long to be compared with high-frequency objective measures of cardiac function. Clinical studies involving digital measures and PROs often face the challenge of aligning high-frequency objective measures collected by digital devices with the low-frequency recall period of some PROs. Patient diaries can, however, be deployed via a smartphone application at a higher frequency than traditional PROs to capture patients’ impression of change [91], and aspects such as symptoms and sleep [92–94]. Symptom trackers [95] and daily PRO instruments are already in use in FD [4–6].

Furthermore, the targeted literature search identified the EQ-5D as being used to assess quality of life in FD [63]. People living with FD have a lower quality of life and reduced functional status compared with the general population [63]. Feedback from patients during the advisory board further supports the notion of lower quality of life and fluctuating functional status of people living with FD. The daily recall period of the EQ-5D allows for sufficient comparison against high-frequency objective measures of quality of life. Patient diaries are critical to track daily or less-frequent changes in symptoms to support care conversations with physicians. Feedback from the patient advisory board about their willingness to complete diaries and questionnaires supports their inclusion in the proposed measurement concept (Fig. 7), although completion times should remain within the timeframes identified to avoid imposing a significant time burden.

Utilizing a patient diary, the KCCQ, and the EQ-5D enables the measures outlined in the measurement concept to be captured. The use of these validated PROs in conjunction with a patient diary meets the concept development criteria for building a digital biomarker as per industry standards (Fig. 2).

## Conclusion

**Fig. 8:**
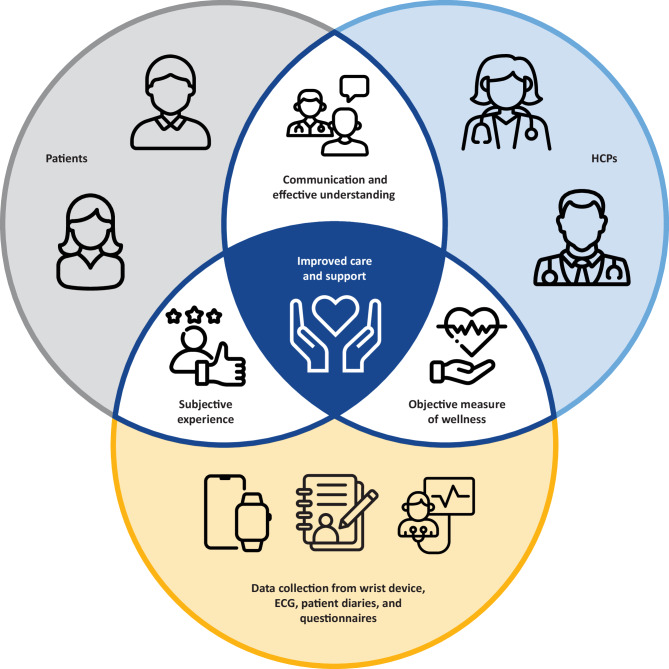
A holistic care approach for improved patient care and support. ECG, electrocardiogram; HCP, healthcare professional

Combining views and input from multiple sources leads to a holistic care approach, with physicians better understanding the experiences of people living with FD, and people with FD increasing their health literacy, ultimately leading to improved care to support better outcomes (Fig. 8). The development of a composite measurement concept that utilizes new and established digital measures to improve the measurement of cardiac function in FD is conceptually possible based on the findings of a targeted literature review, patient advisory board, and clinician advisory board (Fig. 7). The clinical association and concept development work completed to date aligns with the evidentiary framework, V3, and DHT guidance for designing and building a monitoring biomarker that has been described in this paper.

The composite measurement concept outlined here may be used as the foundation for future analytical validation, usability, and clinical validation, seeking to capture the progression of cardiac function in FD.

### Strengths

The strength of this initiative lies in the robust methodology used for concept development, as well as patient and expert support for the critical need of a cardiac monitoring biomarker in FD. Experts agreed on its importance for both clinicians and patients to enable earlier diagnosis and appropriate multisystemic monitoring and management. The need for this initiative is highlighted by the burdensome nature of long in-clinic visits, often requiring costly in-hospital stays, and the lack of a standard protocol for FD care, which pose challenges for tracking disease progression and detecting event risk.

### Limitations

The measurement concept is a preliminary characterization that would need to be followed by an evaluation protocol, then analytical and clinical validation within the context of a supervised clinical study. Any future analytical validation, usability testing, or clinical validation in FD should consider existing work that has been done to date, including the UK National Health Service (NHS) RaILRoAD study [96] and the 2019 review of FD in cardiology practice by Hagège et al. [90].

The extent to which the measurement concept can be generalized warrants further exploration. Although globally based clinicians were involved in the clinical association and concept development work, only a small population of English-speaking, US-based patients were included in this exercise. Any future validation studies should evaluate usability and acceptability by a range of patients with FD, considering factors such as language spoken, age, sex, and degree of functional impairment. It will also be important to assess whether digital tools can be used by, and record data from, patients with significant functional impairment. In addition, the focus on cardiac features in recognition of the significance of cardiac morbidity and mortality in FD may limit generalizability to other FD phenotypes. Moreover, while the literature acknowledges sex-based differences in cardiac features [19, 20, 76], it also suggests that these differences have not yet been qualified using the measures proposed; additional investigation may be required in this respect.

The practicalities of data collection merit additional study. Balancing patient burden in terms of number of devices, ePROs, and completion time while managing collection of enough data points within short timescales will require consideration during implementation of this work. Collecting data for a long enough timeframe to detect meaningful changes in disease progression may also be a challenge. Understanding the extent to which reported symptoms in ePROs are modified by FD-specific treatment or by the disease itself must also be considered. Finally, post-market use would require implementation of additional reporting systems and procedures, and could also include monitoring of cardiac events if validation results support this development step.

## Data Availability

Study data are available from the study sponsor upon reasonable request.
